# Coronavirus Disease 2019 Vaccination for Cancer Patients: Risk or Benefit?

**DOI:** 10.1055/s-0042-1745788

**Published:** 2022-07-12

**Authors:** Bruno Eduardo Pereira Laporte, Estela Gelain Junges Laporte, Paula Fonseca Aarestrup, Matheus Fonseca Aarestrup, Fernando Monteiro Aarestrup

**Affiliations:** 1 Faculdade de Ciências Médicas e da Saúde, Juiz de Fora, MG, Brazil; 2 Universidade Federal de Juiz de Fora, Juiz de Fora, MG, Brazil; 3 Escola de Medicina e Cirurgia, Universidade Federal do Estado do Rio de Janeiro, Rio de Janeiro, RJ, Brazil; 4 Universidade Presidente Tancredo Almeida Neves, São João Del Rei, MG, Brazil; 5 Allergoly and Immunology Department, Hospital Maternidade Therezinha de Jesus, Juiz de Fora, MG, Brazil

**Keywords:** cancer, COVID-19, vaccination, protocols, câncer, COVID-19, vacinação, protocolos

## Abstract

**Objective** The aim of the present study is to list the published clinical trials on coronavirus disease 2019 (COVID-19) vaccines, to describe the mechanism of action of the identified vaccines, and to identify protocols regarding safety, status, and prioritization of cancer patients for vaccination.

**Methods** This is a systematic review with a limited literature search conducted by an information specialist; key resources such as PubMed and websites of major cancer organizations were searched. The main search terms were *COVID-19* , *vaccination* , *cancer* , and *breast and gynecological cancers* .

**Results** Cancer patients infected with the new coronavirus are at high risk of complications and death, but we still know little about the risks and benefits of vaccination for COVID-19 in these patients. In an ideal scenario, all cancer patients should have their immunization status updated before beginning treatment, but this is not always possible.

**Conclusion** Patients with breast or gynecological cancers who are receiving treatment or are in the 5-year posttreatment period should be included in the priority group for severe acute respiratory syndrome coronavirus-2 (SARS-CoV-2) vaccination.

## Resumo

**Objetivo** O objetivo do presente estudo é listar os ensaios clínicos publicados sobre as vacinas para *coronavirus disease* *2019* (COVID-19), descrever seus mecanismos de ação e descrever protocolos sobre segurança, status e priorização de pacientes oncológicos para vacinação.

**Métodos** Trata-se de uma revisão sistemática com uma pesquisa bibliográfica limitada conduzida por um especialista em informação; bases de dados como PubMed e sites das principais organizações de câncer foram pesquisados. Os principais termos de pesquisa foram *COVID-19* , *vacinação* , *câncer* e *câncer de mama e ginecológico* .

**Resultados** Pacientes com câncer infectados com o novo coronavírus têm alto risco de complicações e morte, mas ainda sabemos pouco sobre os riscos e benefícios da vacinação para COVID-19 nesses pacientes. Em um cenário ideal, todos os pacientes com câncer deveriam ter seu estado de imunização atualizado antes de iniciar o tratamento, mas nem sempre isso é possível.

**Conclusão** Pacientes com câncer de mama ou ginecológico em tratamento ou no período pós-tratamento de 5 anos devem ser incluídos no grupo prioritário para vacinação contra *severe acute respiratory syndrome coronavirus-2* (SARS-CoV-2.)

## Introduction

The severe acute respiratory syndrome coronavirus-2 (SARS-CoV-2) was first identified in December 2019 in Wuhan, China. On January 12, 2020, the World Health Organization (WHO) published a series of guidance documents on managing the outbreak caused by this novel virus. Subsequently, on January 30, 2020, the WHO Director-General declared the outbreak a public health emergency of international concern, at the highest level of alarm. 1

The new coronavirus is part of a family of viruses known since 1960; the virus is known for the crown-shaped spike proteins on its surface, which play a critical role in its infectivity. The new variant underwent a zoonotic spillover, becoming capable of infecting humans. This phenomenon is believed to have occurred in a Chinese fish market in the following pathway: from bats, via pangolins (mammals found in the tropical areas of Africa and Asia) as the probable intermediary, to humans. 2

According to the WHO, the SARS-CoV-2 is currently responsible for more than 2 million deaths around the world, with approximately 100 million confirmed cases. 3 More than a year after its emergence, there are no specific treatments to change its course. The global population had to adapt to the disease, medical services had to restructure themselves to continue treating chronic diseases, and COVID-19 became the main topic within all specialties as a new variable to be taken into account in any therapeutic plan.

In this context, vaccines emerged as the main weapon against the SARS-CoV-2 pandemic. Vaccines are already a global reality, and understanding the technologies used is essential for clinical practice and adequate patient management.

## Types of Vaccines

### Inactivated Virus

Inactivated virus technology has been successfully used in several vaccines, such as hepatitis A, polio, and influenza; these vaccines are produced from viral replication in cell cultures. The viruses are subsequently inactivated by chemical or physical agents that render them incapable of replication. This process reduces the immunogenicity of the vaccine, making it necessary to use adjuvants and administer it in several doses to induce a robust immune response. The main commercialized vaccines of this type are CoronaVac, from Instituto Butantan, and Covaxin, from Bharat Biotech. 4 5

### Recombinant Viral Vectors

Recombinant viral vector vaccines use viruses that are genetically modified to produce a specific protein. In the case of the SARS-CoV-2, the spike protein interacts with the angiotensin-converting enzyme 2 (ACE2) receptor and allows the virus to enter the cell. Several vaccines use this approach, including the Oxford University/AstraZeneca vaccine, which uses a recombinant chimpanzee adenovirus (ChAd) as a vector; the Sputnik V vaccine, which uses two different types of recombinant adenoviruses (rAd5 and rAd26); and the Janssen vaccine, which is still undergoing clinical trials. 6 7 8

### Messenger ribonucleic acid (mRNA) Vaccines

Messenger ribonucleic acid (mRNA) vaccines are the result of a new technology and acknowledged as one of the most promising vaccines. A strand of mRNA that encodes a specific antigen of the virus is used, and the body's cells use this information to produce this antigen, which is presented on the surface of the cells where it is recognized by the body as “no self” *,* triggering an immune response mediated by the antibodies and T lymphocytes. This technology is being used in the Pfizer and Moderna vaccines. 9 10 Table 1 outlines the main vaccines that are being developed and/or distributed across the world, their characteristics, advantages, and disadvantages.

**Table 1:** Vaccines

Manufacturer	Technology/Platform	Name	Doses	Advantages	Disadvantages	Efficacy
Sinovac Research and Development Co., Ltd. 4	Inactivated virus	SARS-CoV-2 vaccine (inactivated) CoronaVac	Day 0 + 28	Safe for cancer patients Known technology Storage from 2 to 8°C	Low immunogenicity	50.36%
Bharat Biotech International Limited. 5	Inactivated virus	Whole-virion inactivated SARS-CoV-2 vaccine (BBV152)	Day 0 + 14	Known technology Easy storage	Low immunogenicity	Phase 3 in progress
AstraZeneca + University of Oxford. 6	Viral vector (non-replicating)	ChAdOx1-S - (AZD1222) (Covishield)	Day 0 + 28 or 3 months	Longer gap between doses. One dose protection		70.4%
Gamaleya Research Institute of Epidemiology and Microbiology, Russia. 7	Viral vector (non-replicating	Sputnik V Gam-COVID-Vac (rAd26-S and rAd5-S)	rAd26-S given on day 0 and rAd5-S on day 21	Uses different vectors in two doses		91.6%
Janssen Pharmaceutical. 8	Viral vector (non-replicating)	Ad26.COV2.S	Day 0 or Day 0 +56	Possibility of single dose, ongoing studies		Phase 3 in progress
Pfizer/BioNTech + Fosun Pharma. 9	RNA-based vaccine	BNT162 (3 LNP-mRNAs)	Day 0 + 21	Promising technology. Easy adaptation in case of viral mutation	Risk of accumulation in solid tumors due to the use of lipid capsule Storage at -70°C	95%
Moderna + National Institute of Allergy and Infectious Diseases (NIAID). 10	RNA-based vaccine	mRNA -1273	Day 0 + 28	Promising technology. Easy adaptation in case of viral mutation	Risk of accumulation in solid tumors due to the use of lipid capsule Storage at -20°C	94.1%

## Methods

A systematic review with a limited literature search was conducted by an information specialist; key resources such as PubMed and websites of the major cancer organizations were searched. The main search terms were *COVID-19* , *vaccination* , *cancer* , and *breast and gynecological cancers* . The search was limited to the literature in English language and was conducted between January 20 and 26, 2021. The inclusion criteria were original articles from clinical trials of potential vaccines for COVID-19 and recommendations from the main oncology societies. The exclusion criterion was articles that did not meet the inclusion criteria or had insufficient data.

## Objectives

- To list the published clinical trials on COVID-19 vaccines, focusing on those that will be used in Brazil;
- To describe the mechanism of action of the identified vaccines with the aim of understanding their use in cancer patients;
- To identify protocols regarding safety, status, and prioritization of cancer patients for vaccination used by associations, organizations, and reference hospitals for the treatment of cancer patients.

## Discussion

Due to the limited number of vaccine doses available, governments are selecting priority groups for vaccination according to the risk of infection and/or severity of the disease. One such group consists of patients with malignant tumors, including solid and hematological tumors, and those undergoing hematopoietic stem cell transplantation (HSCT), who are at a high risk of morbidity and mortality if infected with COVID-19. 11 12

Brazil is expected to register 625,000 new cancer cases for each year of the 2020/2022 triennium according to the Brazilian National Cancer Institute (INCA). Among these patients, those with breast and gynecological cancers (cervical, ovarian, and endometrial) stand out. According to the INCA's estimate for 2020, these tumors account for 44.8% of all malignant tumors in women. Due to their high incidence and the fact that the treatment includes surgery, chemotherapy, radiotherapy, endocrine therapy, and targeted drugs, the patients and health professionals have concerns about the safety and effectiveness of vaccines against COVID-19. 13

According to a retrospective cohort study conducted by the INCA, cancer patients infected with the new coronavirus are at a high risk of complications and death, probably due to increased clinical frailty and negative impact of immunosuppressive treatments: 14 to 19% of the patients without cancer versus 18 to 38% of the patients with cancer develop severe complications in the presence of COVID-19, indicating the increased likelihood of severe complications in cancer patients. 14 Al-Quteimat and Amer 15 found that cancer patients treated with surgery or chemotherapy 30 days before being diagnosed with COVID-19 have an increased risk of developing severe complications from the disease. In their meta-analysis, Zarifkar et al. 16 showed that the risk of intra-hospital mortality among patients with both COVID-19 and cancer was five times higher than in the group of patients without cancer, which confirms the unfavorable disease course in this group of patients. Two other studies reported increased mortality by COVID-19 in cancer patients when adjusted for age and sex (hazard ratio, 1.4; 95% confidence interval, 1.0–2.0). 17 18 Another study reported an increased risk of mortality among cancer patients under 50 years of age compared with non-cancer patients of the same age group (relative risk, 5.01; 95% confidence interval, 1.55–16.2). 12

In an ideal scenario, all cancer patients should have their immunization status updated before beginning treatment. It is recommended that inactivated vaccines are administered 2 weeks before the beginning of treatment. Attenuated vaccines should be administered 4 weeks before treatment, not only for safety but also to ensure adequate production of immunoglobulins. However, immunization is often necessary while receiving immunosuppressive drugs. Inactivated vaccines do not present an increased risk of adverse events to immunosuppressed patients. Therefore, these vaccines should be administered, and a second dose may be needed after the treatment has ended to ensure an appropriate immune response. Attenuated vaccines (Bacillus Calmette-Guérin [BCG]; rotavirus; oral polio [OPV]; yellow fever; measles, mumps, and rubella/varicella [MMR/MMRV]; chickenpox; and herpes zoster) should not be given during immunodepression. In cases of moderate immunodepression, the physician responsible for the clinical and epidemiological evaluation determines whether yellow fever, MMR, MMRV, chickenpox, and herpes zoster vaccines should be administered. It is important to emphasize that the immunization of close contacts, family members, cohabitants, and health professionals responsible for the patient's care (according to the schedule for their age group) is essential to reduce the transmission of the virus to cancer patients. 19

To date, there is no report of a live-attenuated virus vaccine available for use in Brazil. The Oxford University/AstraZeneca vaccine consists of an adenovirus vector that has no ability to replicate. CoronaVac is classified as an inactivated virus vaccine, and the Pfizer/BioNTech vaccine uses mRNA technology to stimulate immune response. Therefore, these vaccines should not pose a risk to immunocompromised patients, and, from the outset, the only specific contraindication would be hypersensitivity to any of the components of the vaccines. 20

However, the clinical trials that were conducted did not include cancer patients. Therefore, some issues should be addressed:

- Because RNA vaccines are delivered as lipid carriers, they can accumulate in solid tumor tissues. In addition, absorption of the material by these tumors may reduce the efficacy of this type of vaccine. 21
- Patients in maintenance therapy (rituximab, tyrosine kinase inhibitors) may have reduced response to immunization. 22 23 24
- For patients undergoing chemotherapy, it is not yet known if immunization should be given at the time of cytotoxic drug administration or between cycles. The high variability of available regimens makes it difficult to determine the optimal timing, which remains controversial. 22 23 24
- Patients receiving checkpoint inhibitors may experience an exacerbated immune response, associated with an increased risk of adverse events. For safety, the vaccine should be administered on the same day as the treatment, so that, if necessary, patients receive care and do not have to return to the healthcare service. 22 23 24
- Regarding patients who undergo surgery, it is difficult to distinguish between potential reactions to the vaccine and the organic response to the trauma caused by the surgery. It should be noted that a rise in temperature (38°C) is a common reaction to vaccination and may be confused with postsurgical infection. The recommendation is to perform the immunization approximately 2 weeks before any surgical procedure. 22 23 24
- Vaccination does not exclude the need for social distancing and precautionary measures on the part of the patient and cohabitants. 22 23 24

Table 2 outlines the recommendations on Sars-CoV-2 vaccines for each therapeutic modality of the main associations and reference centers for the treatment of cancer patients.

**Table 2:** Recommendations from the main associations and reference centers for the treatment of cancer patients

**Protocol**	**Cytotoxic chemotherapy**	**Radiotherapy**	**Endocrine therapy**	**Targeted therapy**	**Corticoids**	**Immunotherapy**	**Surgery**	**Vaccination priority**	**Observations**
AC Camargo Cancer Center. 20	When blood counts have recovered to the maximum (end of the cycle). Avoid the day of chemotherapy treatment.		Cleared without restrictions.	Cleared without restrictions, provided blood counts are normal.		Cleared without restriction, provided blood counts are normal.			Information obtained from Pfizer-BioNtech and AstraZeneca-Oxford vaccines
National Comprehensive Cancer Network. 22	Cleared without restrictions. There is no evidence of optimal timing of vaccination during treatment.	Cleared without restrictions	Cleared without restrictions (not a priority when used exclusively by non-metastatic patients)	Cleared without restrictions	May reduce the immune response to vaccination. It is recommended to delay vaccination.	Cleared without restrictions.	Do not vaccinate on the day of the surgery because the vaccine symptoms.	Yes. 1b/c priority group by CDC. Patients under endocrine therapy are not prioritized.	
Memorial Sloan Kettering Cancer Center. 23	First dose between cycles, far apart from the nadir period, or after the end of the treatment if the latter is near completion.	Cleared without restrictions	Cleared without restrictions	Cleared without restrictions	Cleared without restrictions	If possible, 2 weeks before the start of therapy	Cleared without restrictions. Exceptions in cases of splenectomy	Yes. Prioritize by age **not** according to location or presence of metastasis.	Avoid immunotherapy or chemotherapy on 2nd or 3rd day after vaccination (period of increased risk vaccine side effects)
European Society for Medical Oncology. 24	Cleared without restrictions. There is no evidence of the optimal timing of vaccination during treatment.					Potential reduction in vaccine effectiveness. The benefits outweigh the risk.		Yes, especially in patients with hematologic tumors (receiving chemotherapy or with active disease), advanced solid tumors, or history of solid tumor in the last 5 years.	Protein kinase and PARP inhibitors do not have timing restrictions. Proteasome and CDK4/6 inhibitors should be used preferably at the end of the cycle, avoiding the day of chemotherapy treatment.
Cancer Research UK. 25	Optimal vaccination timing is before the start of the treatment. If treatment has already started, the patient should discuss the best timing for vaccination with the physician.	Cleared without restrictions.				Cleared without timing restrictions. Benefits are greater than costs.	Vaccination is recommended 2 weeks before surgery.		
MD Anderson Cancer Center. 26	Cleared. However, the optimal timing for vaccination should be discussed with the physician.					Cleared. However, the optimal timing for vaccination should be discussed with the physician.	Vaccination is recommended 2 weeks after surgery	Yes. Increased COVID-19-related morbidity and mortality in cancer patients.	
Cancer Treatment Centers of America. 27	Safe according to available data. Potential reduction in vaccine effectiveness during cancer treatment. A discussion with the physician is recommended.	Safe according to available data. Potential reduction in vaccine effectiveness.							Use of AstraZeneca Oxford vaccine is restricted in immunocompromised patients.
Sociedade Brasileira de Oncologia Clínica. 28	Cleared, but should be given preferably before the start of the treatment.				Cleared. There is no evidence of the need for a second vaccine dose in immunosuppre-ssed patients.	Cleared. Risk of interference with vaccine response and side effects.		Yes. Increased Covid-19-related morbidity and mortality in cancer patients.	

## Conclusion

Although the evidence regarding the safety of Sars-CoV-2 vaccines in cancer patients is limited, the increased risk of morbidity and mortality of the disease in this group, the well-documented benefit of a vaccination plan for cancer patients, and the theoretical rationale of the mechanism of action of the vaccines already available are sufficient for prioritizing patients with breast or gynecological cancers who are receiving treatment or are in the 5-year posttreatment period for SARS-CoV-2 vaccination. Urgent and effective public policies are needed for this vulnerable group of patients; patients who remain disease free after 5 years should be included in their respective age group for vaccination prioritization. We emphasize that due to the scarcity of data, these recommendations may be modified as new evidence comes to light.
